# Vocal Imitation in Parrots Allows Addressing of Specific Individuals in a Dynamic Communication Network

**DOI:** 10.1371/journal.pone.0049747

**Published:** 2012-11-21

**Authors:** Thorsten J. S. Balsby, Jane Vestergaard Momberg, Torben Dabelsteen

**Affiliations:** Department of Biology, University of Copenhagen, Copenhagen, Denmark; University of Lethbridge, Canada

## Abstract

Parrots in captivity are known for their ability to vocally imitate humans and recently it has been shown that wild-living orange-fronted conures are able to immediately imitate other individuals’ contact calls. The function of this exceptional ability to imitate remains unclear. However, orange–fronted conures live in fission-fusion flocks where they encounter many different individuals every day, and it is possible that their vocal imitation ability is a flexible means to address a specific individual within a flock. We tested this via playback to short-term captive wild conures. Test birds were placed together in pairs in outdoor aviaries to form simple flocks. To simulate imitation of a specific individual these pairs received playback of contact calls that primarily imitate one of the two birds. Overall, individuals that received simulated vocal imitations of its calls responded more frequently and faster than the other individual. This suggests that orange-fronted conures can use imitations of contact calls to address specific individuals of a flock. In the discussion we argue that the fission-fusion flock dynamics of many parrot species has been an important factor in evolving conures’ and other parrots’ exceptional ability to imitate.

## Introduction

Animals often communicate in network environments, in which multiple unintended receivers may be present [1]. The ability to direct signals to specific individuals (addressing) can therefore provide an advantage by affecting only the behavior of the intended receiver and not the whole network.

Mechanisms for addressing individuals include vocally matching aspects of the addressee’s vocalisation, timing vocalisations relative to those of the addressee, and orientating towards the intended receiver [2], [3]. Addressing by vocal matching can be accomplished in two different ways: (1) by using a vocalisation type that resembles the one used by the addressee and which is already part of the repertoire of the addressor, or (2) through vocal imitation in which the addressor alters its vocalization type to resemble the variant of the addressee. Several territorial species perform vocal matching using song types from their repertoires, e.g. great tits (*Parus major*) [4], [5], western medowlarks (*Sturnella neglecta*) [6] and song sparrows (*Melospiza melodia*) [7]. In these species, song type matching by a simulated intruder elicits strong responses from territory owners and thus indicates that matching addresses the matched individual [7], [8].

Whereas call or song type matching is limited by the number of vocalisation types in the repertoire, imitation provides a more flexible mechanism for matching. Vocal imitations of individuals’ distinct vocalisations have the potential to address specific individuals within a communication network; however, such addressing has not been shown experimentally for any species. Vocal imitation of conspecifics’ individually specific vocalisations during interactions in non-territorial contexts has been observed among bottlenose dolphins (*Tursiops truncatus*) [9], [10], [11], galahs (*Eolophus roseicapillus*) [12] and orange-fronted conures (*Aratinga canicularis*) [13], [14]. The flexibility of vocal imitation will enable individuals to uniquely address all conspecifics in a communication network, without requiring prior knowledge of or familiarity with the interacting individuals. Spectacled parrotlets seem to address specific individuals in small stable flocks, which was interpreted as vocal labelling [15]. It is, however, possible that the contact calls of spectacled parrotlets could involve imitations, but that hypothesis has not yet been tested.

In this experiment, we simulate vocal imitation by using variants of contact calls similar to an intended receiver’s contact call to determine if specific receivers can be addressed in a non-territorial communication network. Previous experiments on orange-fronted conures have demonstrated their ability to rapidly imitate contact calls of other individuals [13], [14], and that imitations of contact calls have signal value for receivers [16]. The functional significance of contact call imitation for receivers has not yet been explicitly tested, although this is crucial for understanding the evolution of vocal imitation among conspecifics. Our playback experiment tests the potential of addressing a specific individual in a communication network by vocal imitation. We monitor the response of both intended and non-intended receivers in the communication network. To our knowledge, no experiments have monitored how non-intended receivers in such networks respond, although these individuals may also respond to the stimuli and indirectly affect the response of the focal bird.

Our study species, the orange-fronted conure, communicates in large and dynamic communication networks. Outside the breeding season they are non-territorial and live in flocks with fission-fusion dynamics with frequent changes in flock composition [17]. Flock fusions are always preceded by exchanges of contact calls [13]. The contact calls of the orange-fronted conure show individual and sexual distinctiveness [18], [19], and are uttered both when individuals are alone (solo contact calls) and during vocal interactions. Solo contact calls show very little variation, whereas contact calls given during interactions are more variable (unpublished data). During natural contact exchange and during vocal interactions with playback [13], [14], orange-fronted conures often imitate contact calls by modifying the call’s fine scale structure. The imitations gradually increase the similarity between individuals’ calls, resulting in a convergent interaction [13], [14]. In other interactions, however, orange-fronted conures can decrease the similarity between the contact calls, resulting a divergent interaction. Imitations of contact calls have signal value for orange-fronted conures as imitation elicits high contact calls rates in the receiver [16].

Previous experiments have only tested the signal function of imitation within dyadic interactions with playback to a single subject at a time [16]. In this experiment, we create a simple communication network by joining two wild-caught orange-fronted conures from different flocks in the same aviary and presenting playback stimuli to them simultaneously. Our aim was to determine if specific receivers can be addressed in a non-territorial communication network. To do so, we simulated vocal imitation through playback of variants of contact calls similar to that of an intended receiver. If vocal imitation addresses specific individuals, we expected the imitated individual to be the primary respondent to the playback. Orange-fronted conures respond with most calls in interactions with the opposite sex and where female test-birds generally gave more calls than male test birds. Given the strong influence of sex in other experiments [16], we expected an overall stronger response of female than male test birds. Most experiments on vocal matching have only monitored the vocal response of the focal bird [4], [5], [8], [20] although other individuals of the local network respond after eavesdropping on the experiment [21],[22],[23],[24]. Our experimental setup, however, enabled us to monitor the vocal response of the whole communication network rather than just the focal bird.

## Materials and Methods

We conducted the experiment during the non-breeding season from June 16 to August 6, 2007, at Santa Rosa National Park, Area de Conservación Guanacaste, Costa Rica. We captured orange-fronted conures in mist nests, using playback of contact calls to lure them in. In this experiment we used 36 orange-fronted conures from 27 different flocks. Four aviaries (dimensions: 3.5×1.8×1.8 m) housed the test birds. Aviaries were located in the natural habitat of dense shrubbery at least 300 m apart and were thus considered acoustically isolated from each other. All aviaries provided rain cover and several branches for perching. In the aviary, test birds had unlimited access to water and food (nancite fruits, *Byrsonima crassifolia*). Test birds spent 2–7 days in the aviary. For the first 2–4 days, each test bird was housed individually and their individually distinctive solo contact calls were recorded, which we used to specifically tailor the playback stimuli to the individuals in each trial (see below). Once the solo contact calls for both test birds had been obtained, we moved one of the test birds to the aviary of the other individual for the playback experiment. Because flock mates of some species converge their contact calls [25], [26], [27], we always used birds from different flocks to form our new flocks to ensure that contact calls differed. Although imitation in orange-fronted conures occurs rapidly during an interaction, sustained convergence of individually-specific contact calls is unlikely in the timescale of this experiment.

Orange-fronted conures are sexually monomorphic [28]. Sex was therefore determined molecularly from blood samples extracted from the wing vein after the experiment (primer description [29], [30]). The molecular sexing showed that we had 16 female and 20 male test birds. Of the 18 pairs, three pairs consisted of females, 5 pairs consisted of males and 10 pairs consisted of a male and a female. To enable individual identification, we gave each test bird distinctive marks on breast and/or head using felt pens.

### Playback Stimuli and Solo Contact Calls of Test Birds

The contact calls used for playback had been recorded between mid-June and early-August 2005 and 2006 from 14 male and 10 female orange-fronted conures from the local area that were temporarily held individually in the aviaries. The stimuli calls were recorded using a Marantz PMD 670 or 690 solid state recorder and a Sennheiser 816T MKH microphone placed 0.5 metres from the corner of the aviary, so the distance between the microphone and the test birds never exceeded 4.5 m.

**Figure 1:**
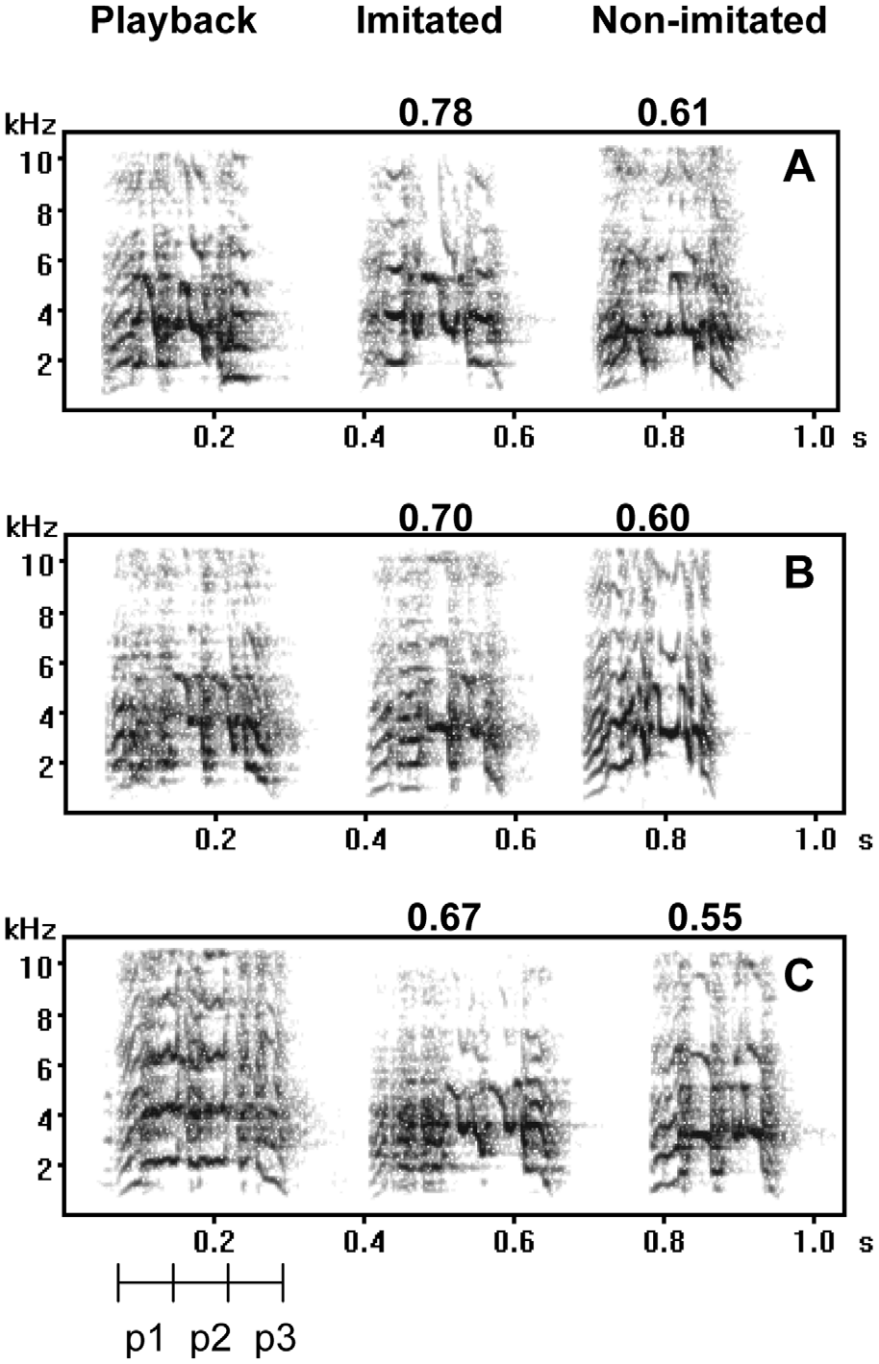
Spectrograms (A–C) of orange-fronted conure contact calls from three different playback trials to different flocks. In each spectrogram the first call is the playback call, the second call is a solo contact call of the target bird (imitated bird) and the third call is a solo call of the non-imitated bird. The three playback calls came from three different flocks. The numbers above the solo contact calls are their cross-correlation similarity relative to the playback call. In A and B the playback call came from a male and in C a female. The test birds in A and C were all females and in B the test birds were both males. The three parts of the contact call are marked below the first call in spectrogram C. The spectrogram has been prepared in Avisoft (FFT = 256, Overlap = 75%, Blackman window).

We recorded contact calls of the test birds between 05∶30–08∶30 am and 03∶00–05∶00 pm using a Marantz PMD 670 solid state recorder and a Sennheiser ME67 K6 supercardioid microphone positioned as described above. We extracted solo contact calls from the recordings using Syrinx version 2.5 s (John Burt http://www.Syrinxpc.com). Acoustic characteristics of the extracted contact calls were measured on spectrograms using a transform size of 512 and a fixed time line of 0.5 s/line. Contact calls can be divided into three segments (Figure 1): a rising harmonic series ascending about 1 kHz over the duration of the segment (P1), a middle section with deep (up to 3 kHz) step-like frequency modulations (P2) where the main part of the energy is between 3 kHz to 6 kHz (P2), and a decreasing harmonics series in which the frequency descends 1 to 3 kHz over the duration of the segment (P3) [17], [18]. For each contact call we measured the length of the entire call and the length of each of the three segments, P1, P2 and P3.

To identify a representative set of individually-distinct solo contact calls for each individual, we chose the 12 most similar solo contact calls given by each test bird, except for three birds from which we only obtained 10 or 11 solo contact calls. The evaluation of similarity was based on the length of the different segments (P1–P3) and the frequency contour of the contact call, in particular P2. Once the 12 most similar solo calls of a test bird were identified, we calculated the mean total call length and mean length of each of the three segments, P1, P2, and P3.

Each trial consisted of a loop with the same stimulus call played back 10 times with 10 second intervals between calls. A stimulus call was chosen from our library of contact calls on basis of its similarity to the contact calls of the test bird, with similarity assessed on the basis of frequency contour and the averaged measures of the 12 solo contact calls described above. Selection was based primarily on the total lengths, secondarily on the lengths and contours of P2, and finally on the length and contours of P1 and P3. We chose these criteria becuase the time parameters of the contact calls contribute most to individual differences among orange-fronted conures [19].

Our aim during playback was to imitate one of the two test birds. Because the two birds inevitably differed in the length of their solo contact calls, we selectively imitated the test bird with the shortest contact call by choosing stimulus calls that were slightly shorter (approximately 5–10 msec) than the average length of the solo contact calls of the test bird. Thus it ultimately made the stimuli even less similar to the non-imitated bird. Similarly, the test bird with the longest solo contact call was imitated by stimulus calls that were longer than the average length of its solo contact calls. The selected stimulus calls were filtered (0.5–11 kHz) and the amplitude standardised with respect to peak amplitude.

Playback experiments have demonstrated that the sex of test birds and stimulus birds influences the response. Female test birds are generally more responsive than male test birds, and heterosexual interactions elicit stronger responses than interactions with the same sex [16]. We therefore included the sex of both test- and stimulus-birds in the design. To account for any effect of sex of the stimulus birds, we imitated each test bird with calls from both a female and a male stimulus bird. Each pair of test birds hence received a total of 4 trials and the playback imitated each of the test birds twice. Within a pair of test birds 4 different stimulus birds were used for the 4 trials.

### Execution of the Playback

All playbacks took place between 5∶30–12∶00 am and between 3∶00–5∶00 pm, which reflect periods of high vocal activity and hence increased likelihood of response to playback. The test birds in each pair were imitated (i.e. became the intended receiver) alternately, and the order of male and female stimuli was randomised. Each playback trial consisted of a playback period, which lasted 101.1±2.7 s (average ± SE) (range of 97.7–108.3 s), and a 5 minutes post-playback period. The playback period started with the first playback and ended 10 seconds after the last contact call in the playback had been played, which defined the beginning of the post-playback period. Because the aviaries are in natural habitat, test subject could interact with free-roaming conspecifics. To reduce any carry-over effects from these interactions, the playback only started if the two test birds had not interacted with free conspecifics for at least 3 minutes. A minimum of 10 minutes elapsed between each of the 4 trials. However, the average time between trials became 13±1 minutes (mean ± SE, range:10–24 minutes) due to interactions with orange-fronted conures outside the aviary.

The stimuli were played back using Syrinx (www.Syrinxpc.com) from a PC (IBM ThinkPad R51 type 1831 model KG5) amplified by a Pioneer GM-3200T amplifier and broadcasted by a JBL Northridge series N24 8 ohms speaker (frequency response: 75–20000 Hz). We placed the speaker in a tree 5–7 m from the center of the aviary and approximately 1.5 m above ground. All playback trials were recorded in the same manner as the contact call recording of individual birds. Furthermore we also videotaped the trials using a Sony DCR HC45 video camera located at one end of the aviary.

We re-recorded the playback calls to account for any minor distortion by the playback equipment. All playback calls were re-recorded at a distance of 7 metres between the speaker and the microphone and 1.5 metres above ground, which was equivalent to the distance between the speaker and the aviary.

### Data Analysis

On basis of the audio recordings of each trial, we extracted and logged the time of every contact call given from the two birds in the aviary. To identify the vocalising individuals, we examined the video recordings of the experiment.

We rejected the trials of the playback or the post-playback periods in the analysis if a) the caller of all contact calls in a period could not be assigned or if b) any of the test birds interacted with birds outside the aviary during the playback period or within the first 2 minutes of the post-playback period. If the playback period was rejected due to interactions with birds outside the aviary, we also rejected the subsequent post playback. On basis of these rules, we used 44 of the 72 trials in the data analysis. For the playback and the post-playback we counted the number of contact calls given and calculated the call rate per minute for each of the test birds. Response latency for each individual (imitated and non-imitated test birds) was defined as the time (in seconds) from the start of the first stimuli call to the beginning of the first contact call given by the individual.

### Success of the Stimulus Selection

To determine how well playback calls imitated the test bird’s solo contact calls, we compared the re-recordings of the playback calls with the solo calls using spectrographic cross-correlation (FFT length: 512 pts, overlap: 87.5%, Blackman window, bandwidth: 500–11000 Hz) in MatLab 7.1 [18], [31]. To improve the performance of the cross-correlation [18] we standardized each sound file to a total duration of 500 ms and adjusted the sound file so the call started 50 ms after the start of the file. The playbacks’ successes in imitating the test birds were quantified as the average cross-correlation similarity for a trial between the playback contact calls and the solo contact calls.

### Ethics Statement

Our research follows the Guidelines for the Treatment of Animals in Behavioral Research and Teaching from the Animal Behaviour Society. For the playback experiment we used short term captive orange-fronted conures. We had a research permit from the Area de Conservación Guanacaste (ACG) for capturing, keeping and conducting playback experiments on orange–fronted conures (Permit number ACG-PI-035-2007). The ACG is the regional authority, which administers research permits. We had CITES permits for exporting the blood samples from Costa Rica (Permit number CR-001-2008) and for importing them to Denmark (Permit number IM 0130-807/08).

### Statistical Analysis

We used mixed and generalised linear mixed models to analyse the data, which enabled us to account for the random effects and the repeated measures in the dataset [32]. To test the success of the playback in imitating one of the test birds, we used a mixed model on the call similarity data with trial number and pair identity as random factors. For the analysis we coded whether birds were given an imitation treatment or not. The model consisted of 3 main factors (± imitation of test bird, test bird’s sex, and stimuli bird’s sex) and their second order interactions.

Using generalised linear mixed models, we tested the effect of imitation on the test birds’ response call rate and latency; pair and trial were included as random factors. The full models consisted of 3 main factors (imitation of test bird, test bird’s sex, and stimuli bird’s sex) and their second order interaction effects. All generalised linear mixed models assumed Poisson distribution and were corrected for over-dispersion [32]. We used least significant differences (LSD) to test for post hoc pairwise differences in the generalised linear mixed models. We only made the post hoc pairwise tests for the interactions where one of the two factors varied. All statistics were performed using proc mixed and proc glimmix [32] in SAS 9.1.3 (SAS Institute Inc., Cary, North Carolina, USA).

## Results

### Treatment Success

**Figure 2:**
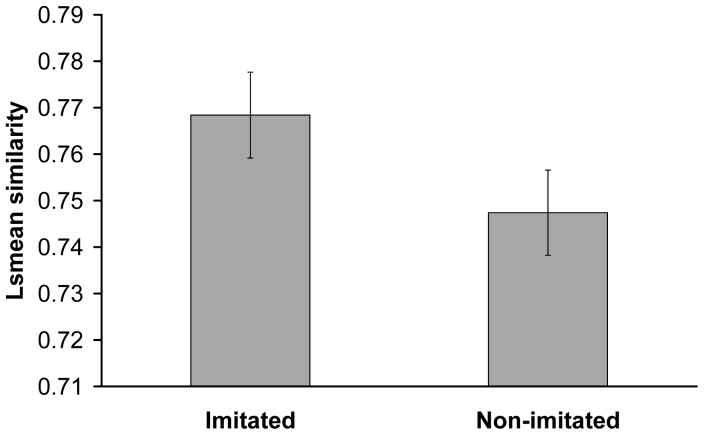
Playbacks successfully imitated the targeted birds. Playback calls (LS mean ± SE) had a higher similarity to the solo calls of imitated than non-imitated test birds. The similarity between the individuals solo contact call and the playback was quantified by spectrographic cross-correlation.

Overall, we obtained a relatively high cross-correlation similarity between playback calls and solo contact calls for both test birds. Solo contact calls from imitated test birds showed significantly higher similarity to the playback calls than those from non-imitated test birds (Figure 1 & 2, Mixed model F_1,39_ = 4.56, p = 0.039). Neither test birds’ sex nor stimuli birds’ sex (mixed model test bird sex F_1,39_ = 0.07, p = 0.79; stimuli birds sex F_1,39_ = 0.04, p = 0.85) or any of the second order interactions involving these factors affected the average similarity values significantly (mixed models all F_1,39_≤0.50, p≥0.48). These results show that the playbacks successfully imitated the targeted test birds. Furthermore, no bias with regard to sex of the test bird or stimulus-bird could be detected.

### Response to Call Imitation

**Figure 3:**
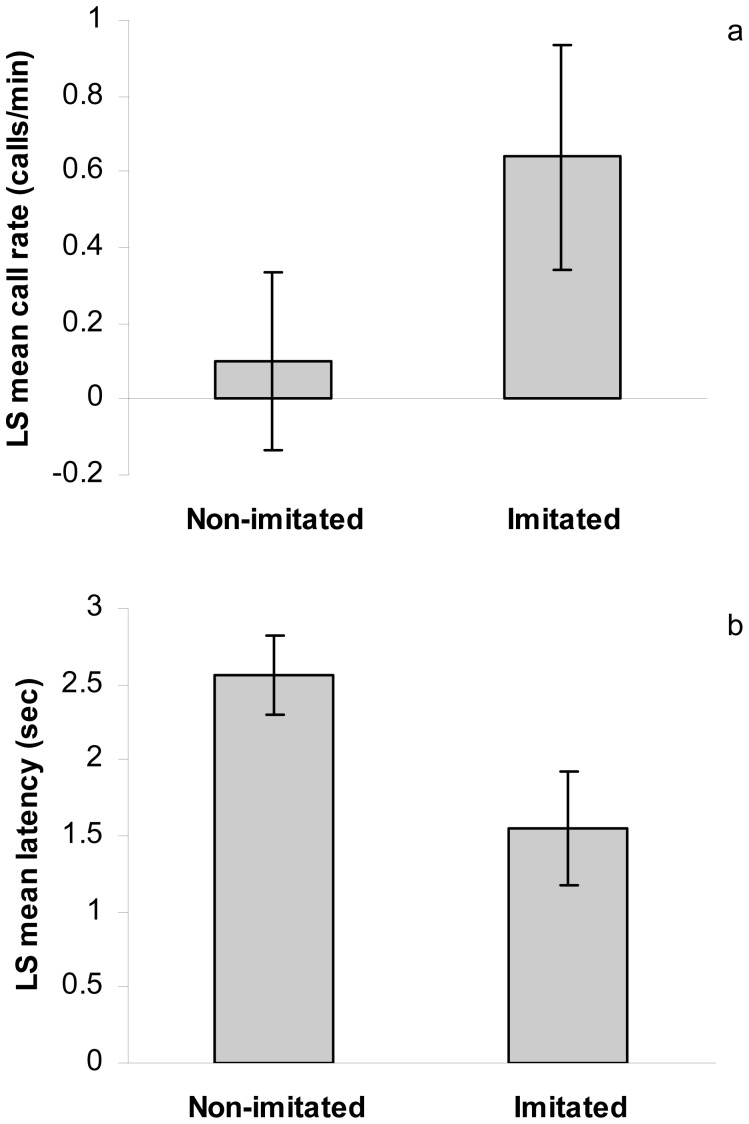
Imitation affected the vocal response. Imitated birds responded with higher call rates and shorter response latency than non-imitated birds. Contact call rates (a) and response latency (b) (LS mean ± SE) of imitated and non-imitated test birds during playback.

Call imitation significantly affected the call rate and latency of the test birds, such that imitated test birds gave significantly higher call rates than non-imitated test birds during playback (Figure 3a; Table 1). Furthermore, imitated test birds showed significantly shorter latency than non-imitated test birds (Figure 3b; Table 1). Imitation had no significant effect on call rate in the post-playback period (Table 1). During playback, the call rate of the imitated and the non-imitated birds were positively related (generalized linear model with random coefficient, intercept = 0.338, t = 1.14, p = 0.270; slope = 0.339, t = 2.30, p = 0.039), such that the non-imitated test birds either responded to the playback or followed the imitated test birds’ response, although at a lower rate.

### Stimulus Birds’ Sex and Test Birds’ Sex

The sex of the stimulus birds did not affect latency or call rates during playback and post-playback (Table 1). Although the call rate during playback to male stimulus birds (2.0±0.5 (mean ±SE)) tended to be lower than that to female stimulus birds (2.4±0.6 (mean ±SE)), this difference was not significant (Table 1). Male and female test birds did not differ in latency and call rate during playback or in call rate during post-playback (Table 1). The interaction between test bird sex and stimulus bird sex showed no significant effect on call rates or latency (Table 1). Hence, our experiment did not detect differences between male and female test birds in the way they responded to stimuli birds of different sex.

### Imitation×Test Birds’ Sex

**Figure 4:**
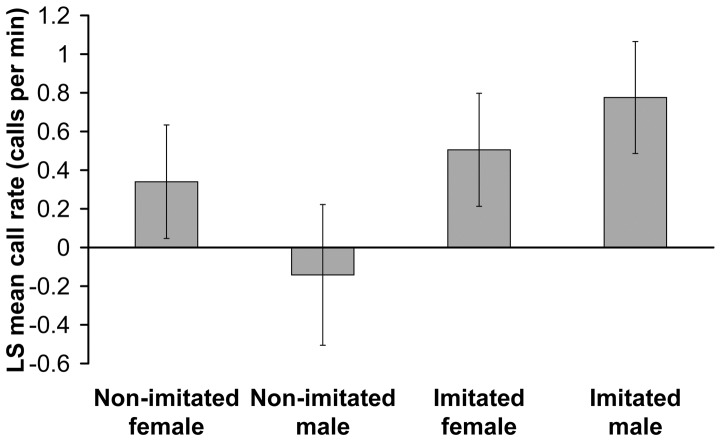
Sex-specific responses to imitation. Males gave higher call rates (LS means ± SE) when imitated than when not imitated, whereas females did not differ in their response to imitation versus non-imitation.

The significant interaction between imitation and test bird sex for the call rate during playback indicated that male and female test birds responded differently when imitated by the playback (Figure 4, Table 1). Post hoc pairwise comparisons showed that males had a significantly higher call rate when imitated by the playback compared to non-imitating playback (Figure 4, least square means t = 3.78, p = 0.0005), whereas the response of imitated and non-imitated females did not differ significantly (Figure 4, least square means t = 0.56, p = 0.58). Although not significant, imitated male test birds tended to respond more than imitated female test birds (Figure 4, least square means t = 1.76, p = 0.09), and non-imitated female test birds tended to call more than non-imitated male test birds (Figure 4, least square means t = 2.01, p = 0.052). The interactions between imitation and test bird sex for latency and post-playback call rate were not significant (Table 1). Likewise the interactions between imitation and stimulus-bird sex showed no significance for any of the response variables (Table 1).

**Table 1:** Responses of test birds.

	Imitation	Test bird sex	Stimulus-bird sex	Imitation×Testbird sex	Imitation×Stimulus-bird sex	Test birdsex×stimulus –bird sex
df	**1**	1	**1**	1	1	1
Latency to calling	**5.98** **(0.027)**	0.01(0.93)	4.23(0.06)	0.01(0.91)	2.55(0.13)	0.19(0.66)
Call rate during playback	**5.61** **(0.023)**	0.09(0.88)	0.08(0.78)	**8.42** **(0.006)**	2.68(0.11)	0.63(0.43)
Call rate post- playback	0.19(0.66)	0.48(0.49)	1.14(0.29)	2.12(0.15)	0.24(0.63)	0.56(0.46)

Generalized linear mixed model on 3 main factors (imitation, test birds’ sex, stimuli birds’ sex), the second order interactions for 3 response variables: latency to calling during playback, call rate during playback, and call rate during post-playback. For each factor and interaction effect the F-value and the p-value (in parentheses) are listed. For call rate during playback and post-playback the degrees of freedom were: df_residual_ = 37, and for latency the degrees of freedom were: df_residual_ = 13.

## Discussion

When multiple orange-fronted conures were simultaneously exposed to the same contact call stimulus, the individual whose call had the highest similarity to the playback call responded the strongest. This suggests that an orange-fronted conure may address a specific individual within a flock by imitating its contact calls. Imitations between calling individuals are common during interactions between free-ranging birds in separate flocks (unpublished data) as well as during individual birds’ responses to playback [13], [14]. Given that orange-fronted conures frequently communicate within large communication networks with many potential receivers, which may be from several different flocks, the ability to selectively address specific individuals may be of particular importance.

Our experiment monitored the response of both intended and non-intended receivers to a sender that did not vary its calls during the interaction. In some natural interactions, the degree of call modification is asymmetric among the interactants; immitation often results from one individual modifying its call more the other individual, rather than a mutual convergence. The playback therefore laid within the natural range of behaviours in orange-fronted conures. Natural interactions between members of flocks would not allow us to discern who addressed who as both flocks (i.e. communication networks) may attempt to address each other. Only an experiment would allow such conclusions. The receiver responses, therefore, are essential for testing whether imitations of contact calls can address other individuals and, ultimately, for understanding the function and evolution of vocal imitation.

Conures can imitate contact calls almost immediately upon hearing them [14], therefore, addressing individuals within a network by imitation does not require any long-term prior experience with specific individuals. This rapid imitation ability is essential, given the fission-fusion flock dynamics that result in a large social network with frequent turnovers in flock composition. In comparison, turnovers in the communication networks of territorial species are less frequent and involve fewer individuals [33]. The relatively small and stable network of territorial species may explain why several of them use song type matching with discrete, existing song types for addressing birds in the neighbourhood [8], [34], [35], [36], [37]. Addressing of specific individuals in a communication network can also be achieved by vocal labeling of individuals, where a specific vocalisation is linked to a specific individual [15], [38], [39]. However, vocal labelling only works in small and/or stable social networks, as it requires prior knowledge of and a learned internal representation of the interacting individuals [40]. Vocal labelling is, therefore, unlikely in large networks with high turnover involving many individuals. In contrast, the plasticity that vocal imitation provides, allows for the addressing of specific individuals with which the addressor has only a limited knowledge. Many species of parrots live part of their lives in social flocks [28] and vocal imitation in parrots may, therefore, have evolved, to enable addressing of specific individuals in communication networks with high turnovers involving many different individuals.

A previous study [16] suggests that convergent contact calls with similarities about 0.6–well below the imitations of the present study (Figure 1 & 2)–are affiliative signals for orange-fronted conures, as male orange-fronted conures call more in response to convergent than to divergent series of calls in a non-agonistic contest [16]. The current experiment provides further insight by revealing that imitations, can be used as a way of addressing individuals. Imitations of the contact calls can thus address and initiate an interaction with a specific individual. Interestingly, males and females responded differently to imitation of their solo contact calls. Males primarily responded more when addressed, whereas females responded both when addressed and when not addressed. Therefore, being addressed seems to be more important to males than to females. In European siskins (Carduelis spinus), which live in flocks part of the year, high ranking males showed mutual hostility towards each other and never made calls that imitated each other. In contrast low-ranking males were less hostile and imitated their flock mates [26]. If imitation of contact calls signals willingness to take a subordinate position in a flock fusion, then the contact call exchanges and imitations after establishing contact by addressing may potentially be used for negotiating dominance position after a flock fusion [16]. Imitation may thus serve two functions: first, it may function in addressing individuals, as demonstrated here. Second, imitation in prolonged interactions may negotiate dominance position after a flock fusion. The strong difference in male and female responses may provide insight to the social organization of flocks in orange-fronted conures. This interpretation suggests that dominance hierarchy/leadership is mainly important to males. Previous experiments support this view [16], since females will respond and attempt to imitate calls with both low and high similarity to their own solo calls, whereas males mainly respond when the playback imitates them. Alternatively, males may respond to imitation because being addressed by imitation is perceived as a challenge. This scenario would be similar to song type matching interactions in passerines [41]. It follows from this interpretation that females do not experience a challenge in the same way by being addressed since they respond to and imitate both convergent and divergent call series. Imitation could thus serve both affiliative and agonistic functions depending on the context and timing of the interaction. Negotiations of dominance relationships in the ensuing interactions may well be agonistic, whereas addressing a specific individual prior to flock fusion would be affiliative.

Many of the experiments that suggest addressing of individuals through song type matching or call imitation have focused solely on dyadic interactions, only monitoring the response of the test subject [8], [14], [34], [35], [36], [37]. However, several experiments have now shown that non-intended receivers within a communication network may extract information from the interactions, i.e. eavesdrop [21], [22], [23], [24], [42], [43]. In this experiment, we created a simple three-member communication network (two live conures, plus playback stimuli) and monitored the response of both the addressed (imitated) and the non-addressed (non-imitated) member of the network. The responses of the addressed and the non-addressed test birds were positively related, indicating that the non-addressed bird attempted to associate either with the playback or the other flock member, although less actively than the addressed bird. This positive relationship is not due to the characteristics of the pair as the statistical model controls for differences between pairs. Our results suggest that future experiments on network communication should monitor the response of the network surrounding the focal individual, if possible.

Vocal pathway development in humans and ancestral birds is believed to have evolved independently [44]. Across taxa, however, several developmental and neural analogies exist such as an innate perceptual predisposition for vocal behavior, and a similar asymmetric brain structure with the left hemisphere specialised for language and song [44], [45]. In parrots, the analogies with humans seem even stronger as both have flexible vocal systems and can learn vocalisations throughout life. A common selection pressure that could give rise to such convergent evolution is the social system; both the hunter-gather life style of early humans [46] and the fission-fusion flock structure of orange-fronted conures [17] result in communication networks that change frequently. Such dynamics within the social networks necessitates flexible communication skills to enable addressing of specific individuals in the network to effectively mediate social interactions within and between groups or flocks. The current study thus demonstrates the use of such plastic communication system in a dynamic social environment, which may explain one reason for the evolution of vocal imitation in parrots.

Orange-fronted conures have the ability to immediately imitate the individually-distinctive contact calls of others. The response to being imitated by a new-comer in a flock context suggests that imitation serves as a way to address specific individuals. The ability to address using imitation probably evolved as a consequence of the complex fission-fusion structure of the orange-fronted conures. Addressing in an ever changing communication network that involve many different individuals necessitated a flexible vocal system to enable addressing of specific individuals in the network.
